# Compare the Quadriceps Activity between Mini-Midvastus and Mini-Medial Parapatellar Approach in Total Knee Arthroplasty with Electromyography

**DOI:** 10.3390/jcm13102736

**Published:** 2024-05-07

**Authors:** Ying-Chun Wang, Sheng-Hua Wu, Chi-An Chen, Jing-Min Liang, Chia-Chi Yang, Chung-Hwan Chen, Wan-Rong Chung, Paul Pei-Hsi Chou, Hsuan-Ti Huang

**Affiliations:** 1 Ph.D. Program in Biomedical Engineering, College of Medicine, Kaohsiung Medical University, Kaohsiung 807, Taiwan; ycwang.ow@gmail.com; 2 Department of Orthopedics, Kaohsiung Medical University Hospital, Kaohsiung Medical University, Kaohsiung 807, Taiwan; hwan@kmu.edu.tw; 3 Department of Orthopedics, Kaohsiung Municipal Ta-Tung Hospital, Kaohsiung Medical University, Kaohsiung 807, Taiwan; 4 Department of Sports Medicine, College of Medicine, Kaohsiung Medical University, Kaohsiung 807, Taiwan; taiga1115@gmail.com; 5 Department of Anesthesiology, Kaohsiung Medical University Hospital, Kaohsiung Medical University, Kaohsiung 807, Taiwan; elsawu2@gmail.com (S.-H.W.); a25628545@yahoo.com.tw (C.-A.C.); 6 Department of Anesthesiology, Kaohsiung Municipal Ta-Tung Hospital, Kaohsiung Medical University, Kaohsiung 807, Taiwan; 7 Department of Anesthesiology, School of Medicine, College of Medicine, Kaohsiung Medical University, Kaohsiung 807, Taiwan; 8 The Master Program of Long-Term Care in Aging, College of Nursing, Kaohsiung Medical University, Kaohsiung 807, Taiwan; chiachiyang@kmu.edu.tw; 9 Center for Long-Term Care Research, Kaohsiung Medical University, Kaohsiung 807, Taiwan; 10 Department of Orthopedics, School of Medicine, College of Medicine, Kaohsiung Medical University, Kaohsiung 807, Taiwan; 11 Orthopedic Research Center, Kaohsiung Medical University, Kaohsiung 807, Taiwan; 12 Regeneration Medicine and Cell Therapy Research Center, Kaohsiung Medical University, Kaohsiung 807, Taiwan; 13 Department of Anesthesiology, E-Da Hospital, Kaohsiung 807, Taiwan; wrchung1228@gmail.com; 14 College of Medicine, I-Shou University, Kaohsiung 807, Taiwan

**Keywords:** total knee arthroplasty, midvastus, parapatellar, electromyography

## Abstract

**Background:** The comparison between the mini-midvastus (mini-MV) and mini-parapatellar (mini-MPP) approach in total knee arthroplasty (TKA) remains a subject of debate. The present study compared quadriceps activation, pain levels, and clinical outcomes between the two approaches; quadricep activation was assessed using surface electromyography (sEMG). **Methods:** This retrospective cross-sectional study comprised a total of 78 patients aged between 50 and 85 years with primary osteoarthritis. Patients were divided into a mini-MV (n = 38) group and a mini-MPP (n = 40) group according to the surgical approach. **Results:** The two groups exhibited no significant differences in sEMG for the vastus medialis (VM) or rectus femoris (RF) at the follow-up time points, with the exception that the mini-MV group exhibited superior strength of RF during extensions at the 2-week follow-up. However, the mini-MPP group had superior Western Ontario and McMaster Universities Index (WOMAC) total and function scores at the 2- and 6-week follow-ups. The mini-MPP group also had superior WOMAC stiffness scores at the 2-week follow-up. The two groups did not differ significantly in terms of pain levels or morphine consumption. **Conclusions:** The sEMG data of quadriceps muscle would not differ significantly between the mini-MV and mini-MPP approaches for TKA. Moreover, the mini-MPP approach may yield superior WOMAC scores when compared with the mini-MV approach.

## 1. Introduction

Total knee arthroplasty (TKA) is highly effective in alleviating pain and restoring functionality in patients with advanced arthritis of the knee joint, particularly osteoarthritis and rheumatoid arthritis. In recent decades, TKA has been widely applied as an intervention for end-stage knee osteoarthritis, with the emphasis being placed on rapid recovery and reduced postoperative pain, which can contribute to overall patient satisfaction [1,2,3]. TKA is frequently undertaken in older adults grappling with osteoarthritis, especially when the consequential pain and functional impairment significantly diminish their quality of life. The surgery involves various controversies, including the selection of implants, approach methods, pain management strategies, and the patient’s inherent activity capabilities, all of which influence recovery times, complication rates, and functional outcomes.

Although several surgical approaches for primary TKA exist, the optimal approach remains controversial [4]. Various approaches have been explored to enhance outcomes and expedite functional recovery [5,6]. Of these approaches, the medial parapatellar (MPP) technique is considered a conventional but effective option. Although the MPP technique provides excellent exposure, concerns persist that intratendinous incisions may disrupt extensor function, resulting in complications such as abnormal patellar tracking and osteonecrosis. By contrast, the midvastus (MV) approach preserves the extensor mechanism and blood supply and mitigates exposure-related drawbacks [7,8,9]. Studies have indicated that the MV approach was associated with reduced postoperative pain, early return of straight-leg raise function, increased range of motion, and decreased need for lateral retinacular release; however, these advantages tended to diminish between 3 weeks and 6 months postoperatively [10,11,12]. The purported advantages of MV over MPP stem from the risk of muscle and innervation damage and remain controversial [13].

Conventional TKA approaches are characterized by extensive exposure and patellar eversion, and these approaches often result in prolonged rehabilitation periods and postoperative pain, which adversely affect patient satisfaction [14,15,16]. The advent of minimally invasive surgery (MIS) in knee arthroplasty, pioneered in the late 1990s, heralded a paradigm shift in surgical techniques. MIS principles were initially applied to unicondylar knee replacement and have since been adopted in TKA, thus offering distinct advantages over standard procedures [17]. Based on current data, MIS TKA offers short-term advantages, such as reduced soft tissue damage, leading to a faster, less painful recovery and a quicker return to normal activities. However, the available evidence suggests that the long-term benefits of minimally invasive total knee replacement do not significantly differ from those associated with traditional total knee replacement. Considering the drawbacks of the conventional MPP approach, scholars have developed modified versions such as mini-MPP to enhance patient outcomes; mini-MPP involves a smaller incision with a limited medial parapatellar arthrotomy extending 2–3 cm into the quadriceps tendon proximal to the patella [5,18]. A study reported that mini-MPP was associated with less blood transfusion and superior flexion in the perioperative period when compared with standard techniques [18]. By contrast, the mini-MV approach is associated with reduced quadriceps injury and improved postoperative results and is thus becoming more widespread. Studies comparing the mini-MV approach with conventional approaches have indicated that the mini-MV approach was superior in terms of postoperative flexion and Knee Society scores [19,20,21]. Although studies have compared mini-MV and mini-MPP approaches, clinical outcomes and radiological findings associated with these minimally invasive primary TKA approaches remain inadequately explored; moreover, the reliability of the findings of such studies is affected by various factors or variables [22,23,24]. Several studies have reported no major differences in outcomes between these approaches, but such studies have not conducted comprehensive evaluations of functional outcomes or quadriceps muscle activation; hence, further research on such outcomes and activation is warranted [25,26,27].

Electromyography (EMG) signals originate from the contraction of skeletal muscles, representing a sequence of potential actions produced by multiple motor units. Through electrodes attached to the skin surface, surface EMG (sEMG) signals can be extracted and studied to assess muscle function in limb movements. sEMG, an instrument systematically developed and refined since the 20th century, finds extensive application in diverse fields analyzing muscle function, including rehabilitation, sports, stomatology, ophthalmology, gynecology, and more [28,29,30]. Accordingly, numerous studies have employed sEMG to capture and analyze motion and muscle activity around the knee joint, particularly focusing on the quadriceps [31,32,33]. The evolution of sEMG from its inception to its current state highlights its transformative impact on medical diagnostics, rehabilitation, and scientific research. Its applications in a wide array of medical disciplines underscore its adaptability and contribute to the ongoing advancements in our understanding of muscle function and its related disorders.

The objective of the present study was to compare quadriceps activation, pain levels, and clinical outcomes between patients who underwent TKA conducted using the mini-MV approach and those who underwent TKA conducted using the mini-MPP approach; quadriceps activation was assessed using sEMG. We hypothesized that the mini-MV approach would result in superior outcomes compared to the mini-MPP approach.

## 2. Materials and Methods

### 2.1. Patient Selection

This retrospective cross-sectional study included patients who received primary TKA at Kaohsiung Municipal Ta-Tung Hospital during the period between February and November 2021. The study protocol was approved by the Institutional Review Board of Kaohsiung Medical University Hospital, Kaohsiung, Taiwan (IRB No. KMUHIRB-E(II)-20230246). The need for written consent was waived. The patient inclusion criteria were as follows: (1) aged between 50 and 85 years and (2) receiving a diagnosis of primary knee osteoarthritis. The exclusion criteria were as follows: (1) having osteonecrosis, (2) having a history of inflammatory arthritis, (3) having a constrained implant applied, (4) having neurological or orthopedic disorders that could affect quadriceps muscle power, or (5) having a history of quadriceps injury. A total of 78 patients met the inclusion criteria and were divided into two treatment groups according to the surgical approach used during TKA: a mini-MV group and a mini-MPP group.

### 2.2. Procedure

All surgical procedures were performed by a single senior orthopedic surgeon (H.-T.H.) at the study hospital; the surgeon used a consistent surgical technique in these procedures. Specifically, the surgeon first created a midline skin incision through the medial third of the patella, starting approximately 2 cm proximal to the upper pole of the patella and extending horizontally to the level of the tibia tubercle. In the mini-MV approach, the median parapatellar retinaculum was then incised from the tibial tubercle level and extended around the medial border of the patella. The deep incision extended proximal to the patella and medially 2–3 cm into the vastus medialis (VM) obliquus muscle [20,34]. In the mini-MPP approach, a limited medial parapatellar arthrotomy was performed with a 2–3 cm division of the quadriceps tendon above the superior pole of the patella, which extended around the medial border of the patella and distally to the tibia tubercle level [5,18]. The patella was subluxed laterally but not everted. Mechanical alignment techniques with proper soft tissue balancing were performed in a standard manner. The same type of posterior-stabilized primary fixed-bearing prosthesis (NexGen LPS-Flex, Zimmer, Warsaw, IN, USA) was implanted with cement and patellar resurfacing in every patient.

All patients received general anesthesia with a laryngeal mask airway and regular multimodal analgesia (MMA) for pain control. After anesthesia induction, the anesthesiologists conducted a single-shot ultrasound-guided adductor canal block with 20 mL of 0.2% ropivacaine. A tourniquet was used during surgery, and the surgeon injected 20 mL of 0.5% bupivacaine and 1 g of tranexamic acid intra-articularly. After the procedure, a fentanyl-based intravenous patient-controlled analgesia (IVPCA) with a security lock duration of 7 min was used to deliver a bolus dose of 10–15 µg over the following 3 days. After surgery, the same oral analgesics were prescribed to all patients: 500 mg of acetaminophen four times a day and 200 mg of celecoxib twice a day for 6 weeks. For thromboembolism prevention, 100 mg of aspirin once daily was prescribed. All patients underwent regular rehabilitation by single physical therapist involving continual passive motion for 30 min twice a day, active quadriceps training for 10 min twice a day, and step training for 5 min once a day from the day after surgery until discharged on the fifth day postoperatively.

### 2.3. EMG Data Collection

An eight-channel portable device (TeleMyo 2400 System, Noraxon, Scottsdale, AZ, USA) was used to obtain sEMG signals from the VM and rectus femoris (RF). After cleansing the skin by using a 70% alcohol swab and cotton to remove dirt and lipids, surface electrodes were positioned along the muscular fibers of the muscle belly. Bipolar circular electrode sensors with a conductive surface diameter of 17 mm (3M™ Foam Monitoring Electrode 2228, Maplewood, MN, USA) were affixed to the skin. We attached EMG electrodes to the leg receiving surgery and collected signals from the quadriceps, including the VM and RF, as described in other studies [35,36]. The patients were briefed on the muscle testing protocol for achieving maximum voluntary contraction (MVC). The patients were examined by a single physician (Y.-C.W.) at designated time points by using three testing methods. The first method entailed evaluating the isometric contraction of the quadriceps; each patient was instructed to lie flat on a bed, fully extend the feet and knees, and perform isometric contractions of the quadriceps five times with maximum force. The second method involved a straight-leg raise test; the patient was instructed to lie flat on a bed, allowing the feet and knees to fully extend, and was then asked to raise the legs five times. The third method involved evaluating knee extension; the patient was instructed to sit on the edge of the bed, with the knees hanging naturally, and was then asked to extend the knees to lift the lower limbs, performing five maximum knee extensions. There was a one-minute interval between each set of repetitions for the subjects. sEMG were conducted in the morning, specifically between 9 and 12 AM. The maximum acceptable electrode input impedance was set to >100 MOhm. The sample rate employed was 1000 Hz. The bandwidth utilized consisted of 1st-order high-pass filters set to a 10 Hz ± 10% cutoff. The common mode rejection ratio was > 100 dB. The input range was ±10 mV. The baseline noise was < 1 uV RMS. The signals were automatically processed. A 100–400 Hz bandpass filter and a 60 Hz notch filter were applied. The parameters for sEMG were configured based on the previously published literature [37,38]. MVC signals from the VM and RF were collected. Data analyses were performed using MyoResearch XP software (Noraxon). The EMG sensors received raw data, after which signals with frequencies of <3 Hz and >1000 Hz were removed. After signal rectification, a 100–400 Hz bandpass filter and a 60 Hz notch filter were applied. A root mean square envelope with a window of 0.125 s was employed. Finally, the MVC data were used to normalize the EMG signals collected in the aforementioned tests, and the peak EMG activation (%MVC) values for each muscle were recorded.

### 2.4. Outcome Assessment

We recorded the patients’ basic demographic data, including age, sex, body mass index (BMI), American Society of Anesthesiologists (ASA) grade, and operating time. Radiological differences were evaluated using the mechanical hip–knee–ankle (HKA) angle. Full-length standing anterior–posterior radiographs of both lower extremities—specifically the hip, knee, and ankle—were taken and analyzed preoperatively and 6 weeks postoperatively at follow-up. The mechanical HKA angle was calculated to represent the change in alignment before and after surgery. Basic demographic data were extracted from medical records, and the HKA data were calculated by a single physician (Y.-C.W.).

The primary outcome comprised between-group differences in peak EMG activation values for each tested muscle under the three testing methods (isometric contractions, straight-leg raises, and knee extensions) preoperatively and on day 1 and day 2 and at 2 weeks and 6 weeks postoperatively. The secondary outcome comprised between-group differences in pain level, fentanyl consumption, and functional outcomes. Pain level was assessed using a numerical rating scale (NRS) on day 1, day 2, and day 3, as well as at 2 weeks and 6 weeks, postoperatively. IVPCA fentanyl dosage was calculated for the first 3 days postoperatively. The Western Ontario and McMaster Universities Index (WOMAC) score was used for functional outcome assessment preoperatively and at 2 weeks and 6 weeks postoperatively [39]. EMG data, functional outcomes, and NRS scores were recorded by a single physician (Y.-C.W.), and morphine consumption was recorded by a single anesthesiologist (S.-H.W.).

### 2.5. Statistical Analysis

Categorical variables are presented as frequencies or percentages and were compared using an χ2 test. All continuous variables were assessed for the assumption of normal distribution using standard tests. Continuous variables are expressed as means ± standard deviations and were compared using a two-sample t-test. All statistical analyses were performed using SPSS (version.19; IBM, Armonk, NY, USA). Statistical significance was set at *p* < 0.05. Cohen’s d classified the effect sizes as small (d = 0.2), medium (d = 0.5), and large (d ≥ 0.8). Sample size estimates were based on the values for quadriceps sEMG activity (the primary outcome variable). A total sample of 72 patients (36 per group) was required (G*Power, version 3.1.9.7). The software settings are configured as follows: test family, t-tests; statistical test, means—difference between two independent means (two groups); and type of power analysis, a priori—compute required sample size-given alpha, power, and effect size. Then, set as two tails, effect size of 0.67, alpha level of 0.05, power of 0.80, and allocation ratio of 1. The effect size of 0.67 is based on our pilot study with a limited number of patients.

## 3. Results

### 3.1. Patient Characteristics

The study included a total of 78 patients, of whom 38 were assigned to the mini-MV group and 40 were assigned to the mini-MPP group. The patients’ demographic and baseline characteristics are presented in Table 1. The mean ages of the patients in the mini-MV and mini-MPP groups were 69.82 ± 7.46 and 72.68 ± 6.42 years, respectively. Female patients constituted a higher proportion of the patients in both groups (86.8% in the mini-MV group and 77.5% in the mini-MPP group). The average BMI values derived for the mini-MV and mini-MPP groups were 27.34 ± 4.79 and 27.24 ± 3.85, respectively. Regarding the distribution of ASA grades in the mini-MV and mini-MPP groups, 34.2% and 37.5% of the patients had a grade II classification, respectively, and 65.8% and 62.5% had a grade III classification, respectively. The mean operating times for the mini-MV and mini-MPP groups were 114.21 ± 15.09 and 109.25 ± 11.69 min, respectively. The preoperative HKA angles for the mini-MV and mini-MPP groups were 7.80° ± 8.66° and 10.37° ± 3.90°, respectively, and the postoperative HKA angles were 0.69° ± 2.58° and 1.69° ± 2.58°, respectively. Accordingly, the two groups did not differ significantly in terms of age, sex, BMI, ASA grade, operating time, or HKA angle.

### 3.2. Pain Intensity

The postoperative pain intensity levels for the patients in the two groups are presented in Table 2. The NRS scores obtained on day 1, day 2, day 3, 2 weeks and 6 weeks postoperatively revealed a gradual amelioration of pain after surgery. The NRS scores obtained at these time points did not differ significantly between the two groups.

### 3.3. Fentanyl Consumption of IVPCA

Data on the patients’ fentanyl consumption through IVPCA are presented in Table 3. The two groups did not differ significantly in terms of daily fentanyl dosage in the 0–12-, 12–24-, 24–48-, or 48–72-h period postoperatively. Additionally, the two groups did not differ significantly in terms of cumulative fentanyl dosages over the first 3 days postoperatively. The two groups did not differ significantly in terms of body weight-based daily fentanyl dosage in the 0–12-, 12–24-, 24–48-, or 48–72-h period postoperatively or cumulative fentanyl dosages over the first 3 days postoperatively.

### 3.4. Functional Results

The mean WOMAC scores for the two groups are presented in Table 4. At baseline, the mean WOMAC pain, stiffness, function, or total scores did not differ significantly between the two groups. At the 2-week follow-up, no significant differences in WOMAC pain scores were observed between the groups, but the WOMAC stiffness (*p* = 0.000), function (*p* = 0.004), and total (*p* = 0.007) scores were significantly better in the mini-MPP group compared with the mini-MV group. At the 6-week follow-up, no significant changes in WOMAC pain or stiffness scores were observed between the groups, but the WOMAC function (*p* = 0.015) and total (*p* = 0.042) scores were significantly better in the mini-MPP group. However, all mean WOMAC scores gradually improved from baseline to the 2- and 6-week follow-up time points.

### 3.5. EMG Results

**Figure 1:**
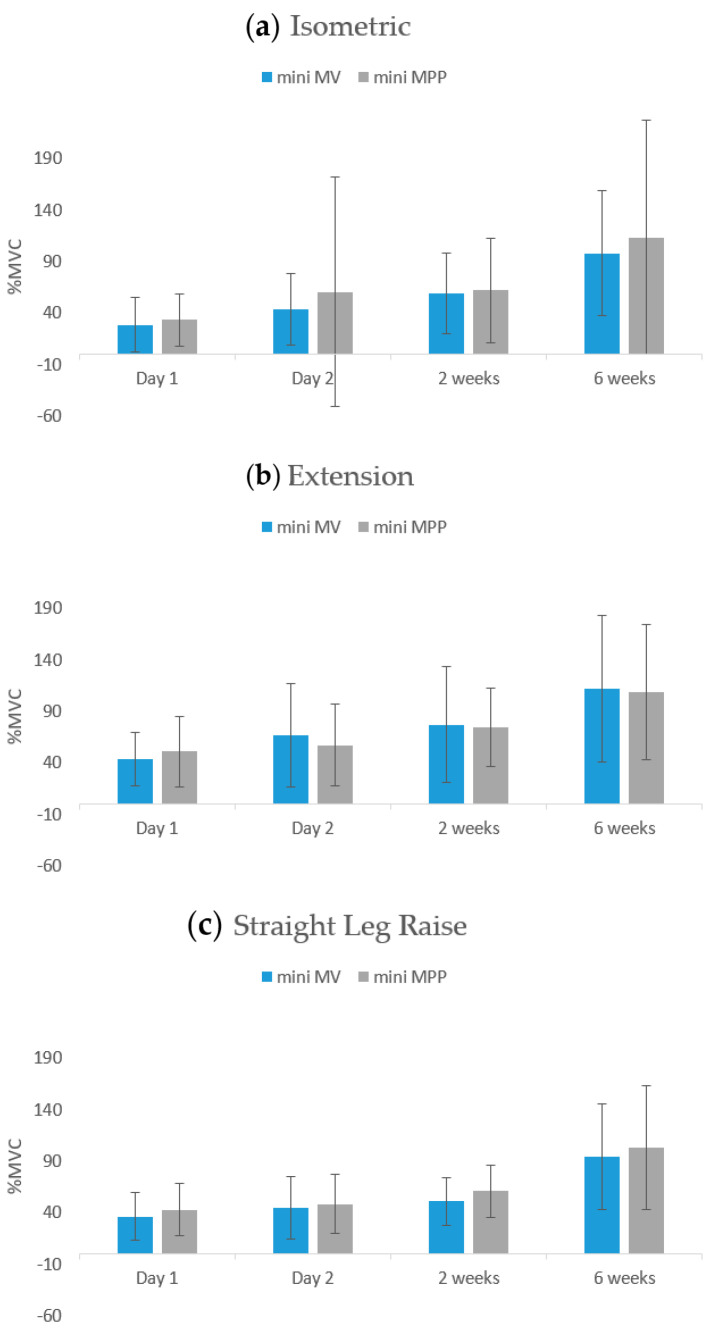
sEMG data of vastus medialis during different movements at different follow-up time points. Error bars indicate one standard deviation.

**Figure 2:**
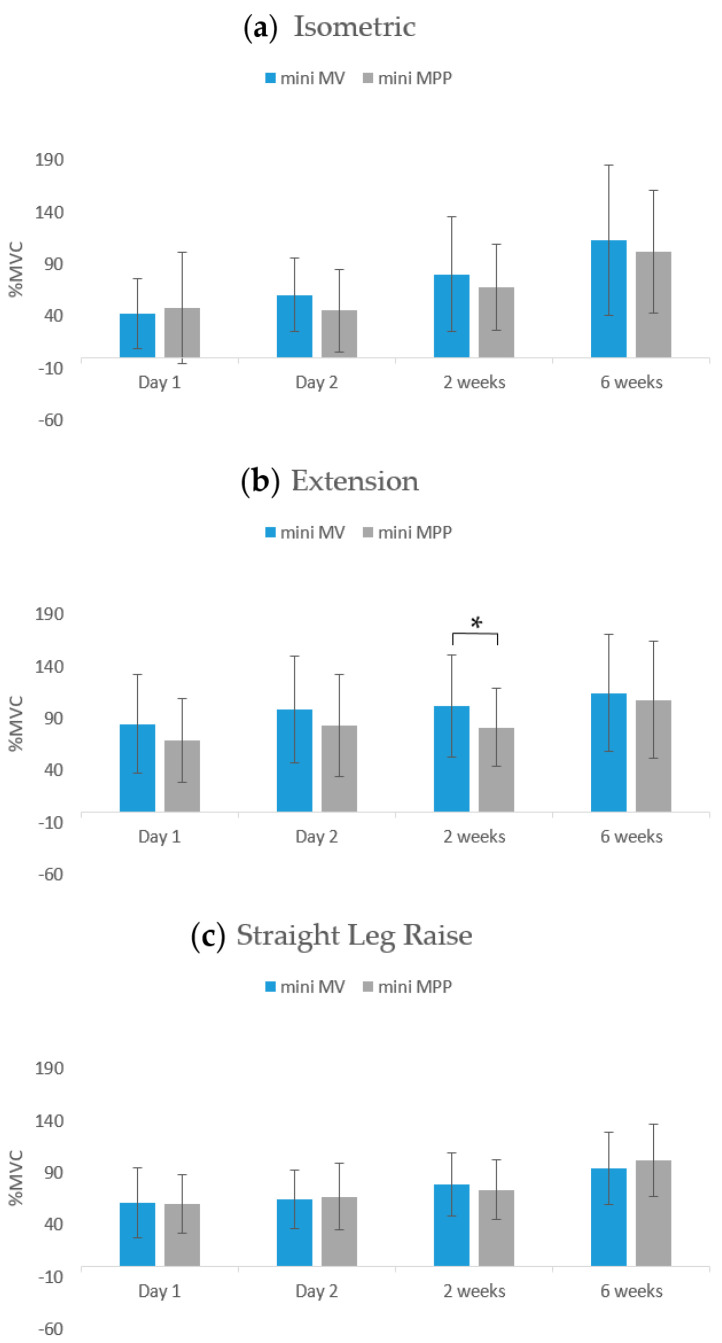
sEMG data of rectus femoris during different movements at different follow-up time points. Error bars indicate one standard deviation. * *p*-value < 0.05.

Regarding the VM EMG data, no significant differences were observed between the two groups in the three movement tests (isometric contractions, knee extensions, and straight-leg raises) on day 1 and day 2 and at 2 weeks and 6 weeks postoperatively (Table 5, Figure 1). Regarding the RF EMG data, no significant differences were noted between the two groups in isometric contractions or straight-leg raises on day 1 and day 2 or at 2 weeks and 6 weeks postoperatively. However, the mini-MV group exhibited superior muscle activation in knee extensions at the 2-week follow-up time point compared with the mini-MPP group (mini-MV: 101.98 ± 48.91 vs. mini-MPP: 81.61 ± 37.73, *p* = 0.042; Table 6 and Figure 2).

No adverse events (thromboembolism, infection, revision, and patella subluxation) were reported during the 6-week follow-up period in any of the patients.

## 4. Discussion

Our sEMG results reveal that the mini-MV group exhibited superior strength recovery of RF when compared with the mini-MPP group during knee extensions at the 2-week follow-up time point only. The two groups exhibited no significant differences in sEMG results for the VM or RF muscles at the follow-up time points. However, the mini-MPP group had superior WOMAC total and function scores at the 2- and 6-week follow-up time points. The mini-MPP group also had superior WOMAC stiffness scores at the 2-week follow-up time point. The two groups did not differ significantly in terms of pain levels or morphine consumption. To the best of our knowledge, this study is the first to use sEMG data to compare short-term recovery outcomes between patients receiving mini-MV and mini-MPP TKA.

The literature indicates that quadriceps strength and pain are the primary factors influencing joint recovery following TKA [40]. Moreover, the link between quadriceps weakness and pain, which can affect disability levels, patient satisfaction, and overall quality of life, is well established [41,42,43]. Therefore, improving quadriceps strength is vital to enabling patients to resume their daily activities. Accordingly, this study examined outcomes related to quadriceps recovery and pain management following TKA.

The decline in quadriceps strength following TKA stems from a well-documented combination of factors, such as pre-existing quadriceps weakness due to knee osteoarthritis, surgical trauma during the TKA procedure, and age-related limitations in muscle function recovery [5,44,45]. TKA provides reliable pain reduction and functional improvement in older adults with knee osteoarthritis; nevertheless, research has indicated that the quadriceps strength may decrease by >50% of its preoperative levels within the first month after TKA [46,47]. This substantial postoperative weakness underscores the necessity of rehabilitation programs to strengthen the quadriceps and emphasizes the importance of investigating the optimal approach to sparing the quadriceps during TKA. One study suggested that the conventional MPP approach may lead to a reduction in quadriceps strength (as measured by isokinetics) of as much as 30.7% over the 2 years following TKA. Moreover, damage to the extensor mechanism may become permanent [48]. In a study evaluating the preoperative and postoperative muscle strength of patients undergoing TKA conducted through the mini-MV approach, rapid recovery times were observed, with patients regaining their preoperative quadriceps strength rapidly. Notably, these levels surpassed those of their preoperative strength by 30% at the 3–6-month time point [11,12]. Some studies have suggested that the ability to perform a postoperative straight-leg raise indicates the recovery of quadriceps strength [49,50]. Specifically, studies have demonstrated that the mini-MV approach enables substantially greater quadriceps strength recovery at 6 weeks, as measured by an earlier ability to perform straight-leg raises [8,9]. Furthermore, several studies have reported that patients undergoing mini-MV procedures demonstrated a considerably earlier ability to perform straight-leg raises [9,26,51]. However, another study found no significant difference in the time before patients could perform straight-leg raises [52]. In one meta-analysis, the mini-MV approach was associated with earlier straight-leg raising times compared with the mini-MPP approach during early postoperative periods [53].

In the present study, in addition to assessing straight-leg raises, we assessed isometric contractions and knee extension movements to evaluate the status of the quadriceps comprehensively. Our sEMG results indicate that although postoperative strength levels were diminished compared with preoperative levels, straight-leg raises could be achieved as soon as the first day after surgery. Moreover, muscle strength was nearly restored to preoperative levels within 6 weeks after surgery. Similar results were observed for both isometric contractions and knee extensions. Although a previous study raised concerns about VM muscle injury under the mini-MV approach, that study did not use EMG data for assessment [54]. Our sEMG results reveal no muscle denervation. Accordingly, the mini-MV approach appears to be a safe form of TKA that does not damage the VM muscle.

Since the advent of the mini-MV approach, variations on the traditional MPP approach have been extensively discussed and validated in the literature. A previous study reported that patients undergoing the mini-MV approach regained motion sooner and exhibited greater range of motion (ROM) in a short-term follow-up period compared with those undergoing the conventional MPP approach [8]. However, the sustained effects on various patient outcomes have not been consistently demonstrated beyond 1 month [8,10,55]. A meta-analysis comparing minimally invasive approaches and the standard MPP approach revealed no differences in hospital-stay length between these approaches; however, mini-MV resulted in superior ROM and straight-leg raises during early postoperative periods [53]. The mini-MPP approach is also associated with positive postoperative outcomes because it can reduce intratendinous disruption. In a simultaneous bilateral TKA study, greater improvement in ROM was observed at 1 week postoperatively in a mini-MPP group compared with a conventional MPP group [56]. Several comparative studies have evaluated the outcomes of mini-MV versus mini-MPP [22,23,24]. Nevertheless, these studies do not recommend one procedure over the other. By contrast, other comparative analyses have indicated that the advantages of the mini-MV approach were limited at best in terms of overall function, muscle strength, pain levels, and other parameters [7,11]. Another study reported no significant differences between mini-MV and mini-MPP groups in terms of functional assessment, patient satisfaction, postoperative complications, quadriceps strength, incisional pain, soft tissue release, or ROM. Moreover, no substantial distinctions were observed between the two groups regarding perioperative parameters or radiographic component positioning [57]. A meta-analysis found that the mini-MV and mini-MPP MIS TKA approaches yielded equivalent clinical outcomes; the mini-MV approach was associated with a significantly longer operating time and higher mean blood loss compared with the mini-MPP approach, but the two approaches did not differ significantly in terms of clinical parameters [58]. Both approaches offer distinct advantages and limitations. The mini-MPP provides excellent exposure and visualization, while also reducing the risk of patellar complications, such as fracture or instability. However, it is associated with the potential for quadriceps fibrosis, which may impact postoperative function. On the other hand, the mini-MV minimizes soft tissue trauma, preserves the quadriceps muscle, and reduces blood loss during surgery. Nevertheless, it may have limitations such as limited exposure and the risk of patellar subluxation. Overall, the choice between the approaches depends on various factors, including the surgeon’s preference, patient anatomy, and pre-existing conditions.

Comprehensive MMA regimens have become the standard of care for patients undergoing TKA. The MMA protocol employed at the study hospital entails integrating oral medications, peripheral nerve blocks, and intra-articular injections to reduce opioid requirements, thus reducing the risks of opioid dependency and other adverse events. The MMA for TKA has not only been proven to decrease opioid consumption but also to reduce pain intensity, increase satisfaction levels, enhance early functional recovery, and shorten hospital stays. In this study, both the mini-MPP and mini-MV groups adhered to identical pain-control regimens involving MMA. The outcomes of this study underscore the efficacy of MMA for post-TKA pain relief, as both groups exhibited consistently low pain scores throughout the follow-up period. Moreover, no significant differences were observed in fentanyl consumption through IVPCA between the two groups over the follow-up period, indicating comparable pain management outcomes. Regarding differences in pain in the present study, no difference in morphine consumption was observed within 3 days after surgery between the mini-MV and mini-MPP groups. No between-group difference in pain levels was observed on day 1, day 2, and day 3, as well as at 2 weeks and 6 weeks, postoperatively; this finding is consistent with the results of previous studies.

However, we observed that the mini-MPP group exhibited superior WOMAC total and function scores at the 2-and 6-week follow-up time points when compared with the mini-MV group; additionally, the mini-MPP group exhibited superior WOMAC stiffness scores at the 2-week follow-up time point. These WOMAC data are inconsistent with the results of previous studies.

These results thus indicate that both mini-MV and mini-MPP are effective and safe for implementation in TKA. Our study is the first to provide robust sEMG data confirming that there is no significant difference in short-term recovery outcomes between patients undergoing the two approaches. Accordingly, we recommend that surgeons employ the most familiar method for minimizing soft tissue damage during TKA.

This study has some limitations. First, we did not use a double-blind design, potentially introducing bias into the group assessments. Second, the sample size was small, and the postoperative follow-up period was short. Third, given the retrospective design of our study, there is a potential for selection bias, and the reliability of our findings is compromised by the dependence on the accuracy of medical records. Fourth, the rehabilitation protocols are based on the practices of the authors’ hospital, but they do not represent the optimal rehabilitation model. Fifth, the sEMG sensors were not placed according to the SENIAM (Surface ElectroMyoGraphy for the Non-Invasive Assessment of Muscle) guidelines. Consequently, larger-scale trials are necessary to provide more robust and comprehensive insights.

## 5. Conclusions

The sEMG data of quadriceps muscle would not differ significantly between the mini-MV and mini-MPP approaches for TKA if intratendinous disruption of the quadriceps is reduced. Moreover, the mini-MPP approach may yield superior WOMAC scores when compared with the mini-MV approach.

## Figures and Tables

**Table 1:** Baseline characteristics of the study population.

	Mini-MV (n = 38)	Mini-MPP (n = 40)	*p*-Value
**Age** (year)	69.82 ± 7.46	72.68 ± 6.42	0.073
**Gender**			0.283
Male, n (%)	5 (13.2%)	9 (22.5%)	
Female, n (%)	33 (86.8%)	31 (77.5%)	
**BMI**	27.34 ± 4.79	27.24 ± 3.85	0.922
**ASA grade**			0.762
II	13 (34.2%)	15 (37.5%)	
III	25 (65.8%)	25 (62.5%)	
**OP Time** (mins)	114.21 ± 15.09	109.25 ± 11.69	0.108
**HKA angle** (°)			
Pre-OP	7.80 ± 8.66	10.37 ± 3.90	0.099
Post-OP	0.69 ± 2.58	1.69 ± 2.58	0.093

MV: midvastus. MPP: medial parapatellar. BMI: body mass index. ASA: American Society of Anesthesiologists. OP: operation. HKA: hip–knee–ankle.

**Table 2:** NRS scores after surgery.

	Mini-MV (n = 38)	Mini-MPP (n = 40)	*p*-Value	Cohen’s d
Day 1	2.24 ± 0.68	2.25 ± 0.54	0.925	−0.016
Day 2	2.24 ± 0.63	2.33 ± 0.76	0.582	−0.128
Day 3	1.74 ± 0.79	1.63 ± 0.87	0.555	0.132
2 weeks	1.68 ± 0.81	1.50 ± 0.75	0.300	0.230
6 weeks	1.39 ± 0.97	1.50 ± 0.88	0.617	−0.118

NRS: Numerical rating scales. MV: midvastus. MPP: medial parapatellar.

**Table 3:** Fentanyl consumption in IVPCA after surgery.

	Mini-MV (n = 38)	Mini-MPP (n = 40)	*p*-Value	Cohen’s d
**Daily dose**				
0–12 h	111.74 ± 61.71	89.53 ± 74.12	0.154	0.325
12–24 h	128.05 ± 128.01	147.73 ± 106.32	0.462	−0.167
24–48 h	199.05 ± 117.86	196.15 ± 121.45	0.915	0.024
48–72 h	162.05 ± 141.05	137.96 ± 96.76	0.385	0.199
**3-Day Total Dose**	600.89 ± 340.55	571.36 ± 273.42	0.673	0.095
**Daily Dose/weight**				
0–12 h	1.70 ± 0.89	1.34 ± 0.97	0.090	0.386
12–24 h	1.94 ± 1.87	2.24 ± 1.58	0.439	−0.173
24–48 h	3.03 ± 1.58	2.99 ± 1.86	0.925	0.023
48–72 h	2.48 ± 1.89	2.06 ± 1.33	0.266	0.257
**3-Day Total Dose/weight**	9.15 ± 4.59	8.63 ± 3.91	0.596	0.121

MV: midvastus. MPP: medial parapatellar.

**Table 4:** Western Ontario and McMaster Universities Index.

	Mini-MV (n = 38)	Mini-MPP (n = 40)	*p*-Value	Cohen’s d
**Baseline**				
Pain	9.24 ± 3.63	8.23 ± 3.17	0.193	0.296
Stiff	3.84 ± 1.39	3.53 ± 1.63	0.359	0.204
Function	22.76 ± 9.67	19.65 ± 9.64	0.159	0.322
Total	35.84 ± 13.55	31.40 ± 12.84	0.142	0.336
**2 weeks**				
Pain	3.18 ± 1.52	3.28 ± 2.01	0.823	−0.056
Stiff	3.32 ± 0.84	2.40 ± 1.17	0.000 **	0.903
Function	19.42 ± 6.61	14.85 ± 6.93	0.004 **	0.674
Total	25.92 ± 7.90	20.53 ± 9.30	0.007 **	0.624
**6 weeks**				
Pain	1.32 ± 1.16	1.58 ± 1.50	0.398	−0.193
Stiff	1.92 ± 0.67	1.63 ± 0.74	0.069	0.410
Function	11.21 ± 6.19	8.05 ± 4.98	0.015 *	0.562
Total	14.45 ± 7.11	11.25 ± 6.57	0.042 *	0.467

MV: midvastus. MPP: medial parapatellar. * *p* < 0.05. ** *p* < 0.01. *p* < 0.05 indicates statistical significance.

**Table 5:** VM.

	Mini-MV (n = 38)	Mini-MPP (n = 40)	*p*-Value	Cohen’s d
**ISO**				
Day 1	28.52 ± 26.58	33.41 ± 25.54	0.411	−0.187
Day 2	43.46 ± 34.78	60.26 ± 111.55	0.377	−0.203
2 weeks	59.30 ± 39.18	62.18 ± 50.83	0.781	−0.063
6 weeks	98.05 ± 60.53	113.74 ± 113.46	0.452	−0.172
**EXT**				
Day 1	43.95 ± 25.96	51.21 ± 34.22	0.296	−0.239
Day 2	66.32 ± 50.24	57.34 ± 40.16	0.385	0.197
2 weeks	76.85 ± 56.25	74.30 ± 38.01	0.815	0.053
6 weeks	112.28 ± 71.03	109.00 ± 66.01	0.833	0.047
**SLR**				
Day 1	36.22 ± 23.17	42.83 ± 25.16	0.232	−0.273
Day 2	45.23 ± 30.38	48.22 ± 28.65	0.656	−0.101
2 weeks	51.02 ± 23.52	61.12 ± 25.38	0.073	−0.412
6 weeks	94.26 ± 51.58	103.68 ± 60.22	0.462	−0.168

MV: midvastus. MPP: medial parapatellar. ISO: isometric. EXT: extension. SLR: straight-leg raise.

**Table 6:** RF.

	Mini-MV (n = 38)	Mini-MPP (n = 40)	*p*-Value	Cohen’s d
**ISO**				
Day 1	42.67 ± 33.36	48.49 ± 53.55	0.568	−0.130
Day 2	60.68 ± 35.08	45.63 ± 39.90	0.082	0.400
2 weeks	80.44 ± 55.42	68.06 ± 41.31	0.265	0.253
6 weeks	113.13 ± 72.64	102.10 ± 59.23	0.464	0.166
**EXT**				
Day 1	84.86 ± 47.72	69.38 ± 40.29	0.125	0.350
Day 2	98.80 ± 51.36	82.96 ± 49.07	0.168	0.315
2 weeks	101.98 ± 48.91	81.61 ± 37.73	0.042 *	0.466
6 weeks	114.84 ± 56.04	108.20 ± 56.51	0.604	0.117
**SLR**				
Day 1	61.58 ± 33.60	59.94 ± 27.90	0.815	0.053
Day 2	64.48 ± 28.08	67.29 ± 32.02	0.682	−0.093
2 weeks	78.91 ± 30.10	73.72 ± 28.86	0.439	0.176
6 weeks	94.55 ± 35.13	102.57 ± 34.75	0.314	−0.229

MV: midvastus. MPP: medial parapatellar. ISO: isometric. EXT: extension. SLR: straight-leg raise. * *p* < 0.05. *p* < 0.05 indicates statistical significance.

## Data Availability

The data presented in this study are available upon request from the corresponding author. The data are not publicly available because of confidentiality issues.
